# Drug Export Pathway of Multidrug Exporter AcrB Revealed by DARPin Inhibitors

**DOI:** 10.1371/journal.pbio.0050007

**Published:** 2006-12-26

**Authors:** Gaby Sennhauser, Patrick Amstutz, Christophe Briand, Otso Storchenegger, Markus G Grütter

**Affiliations:** 1 Department of Biochemistry, University of Zurich, Zurich, Switzerland; 2 Molecular Partners AG, Zurich, Switzerland; Brandeis University, United States of America

## Abstract

The multidrug exporter AcrB is the inner membrane component of the AcrAB-TolC drug efflux system in Escherichia coli and is responsible for the resistance of this organism to a wide range of drugs. Here we describe the crystal structure of the trimeric AcrB in complex with a designed ankyrin-repeat protein (DARPin) inhibitor at 2.5-Å resolution. The three subunits of AcrB are locked in different conformations revealing distinct channels in each subunit. There seems to be remote conformational coupling between the channel access, exit, and the putative proton-translocation site, explaining how the proton motive force is used for drug export. Thus our structure suggests a transport pathway not through the central pore but through the identified channels in the individual subunits, which greatly advances our understanding of the multidrug export mechanism.

## Abstract 2

The high-resolution crystal structure of trimeric AcrB solved using DARPin inhibitors reveals insights into the drug export mechanism via hemi-channels in each subunit.

## Author Summary

Bacterial resistance to antibiotics is a major challenge for the current treatment of infectious diseases. One way bacteria can escape destruction is by pumping out administered drugs through specific transporter proteins that span the cell membrane. We used designer proteins that bind to and stabilize proteins of interest in order to study the major drug efflux pump of *Escherichia coli,* AcrB. After selecting for designed ankyrin repeat proteins (DARPins) that inhibit this pump, we determined the crystal structure of a DARPin inhibitor in complex with AcrB. We confirmed that the AcrB is split into three subunits, each of which exhibits distinctly different conformations. Moreover, we show that each subunit has a differently shaped substrate transport channel; these variable channels provide unique snapshots of the different conformations adopted by AcrB during transport of a substrate. The structure also offers an explanation for how substrate export is structurally coupled to simultaneous proton import—thus significantly improving our understanding of the mechanism of AcrB. This is the first report of the selection and co-crystallization of a DARPin with a membrane protein, which demonstrates the potential of DARPins not only as inhibitors but also as tools for the structural investigation of integral membrane proteins.

## Introduction

Drug resistance is a medical problem, ranging from cancer cells evading chemotherapy to bacteria surviving antibiotic treatment. Efflux pumps represent one class of integral membrane transport proteins in bacteria that confer antibiotic resistance [1]. These proteins actively detoxify the intracellular space by exporting drugs to the cell exterior. AcrB of Escherichia coli is such an efflux pump belonging to the subclass of resistance-nodulation-cell division transporters, which catalyze drug export driven by proton antiport [2]. AcrB associates with the outer membrane channel TolC [3] and the periplasmic protein AcrA [4] and allows direct and efficient transport of a wide range of toxic substances [5]. The structures of AcrB alone [6] and of AcrB in complex with substrates [7,8] revealed the general architecture of the transporter. However, despite all mutational and structural studies to date, the mechanism explaining how substrates are transported into the extracellular media was still unclear.

The use of antibody fragments as crystallization aids for membrane proteins has yielded a number of crystal structures [9,10]. The binding of such antibody fragments enlarges the hydrophilic extramembranal surface of integral membrane proteins, thereby providing additional surface for crystal contacts. They can also stabilize a specific conformation supporting the crystallization process. The drawback of the antibody fragment approach is that it is not always easy to get an antibody fragment that recognizes and binds to a particular conformation of a membrane protein. Further, the selected antibody fragment might be unstable or production might be difficult. To circumvent these problems, we applied an approach based on designed ankyrin-repeat proteins (DARPins) as an alternative to antibody fragments. DARPins can be selected to bind almost any given target protein with high affinity and specificity [11]. They are very stable and can be produced as soluble proteins in large amounts by bacterial expression. As DARPins interact with their target protein with an exposed interaction surface, they tend to bind to conformational epitopes rather than to peptidic ones. These characteristics make DARPins ideal tools to help the structural studies of membrane proteins.

Here we selected DARPins that not only bind to AcrB but also inhibit bacterial drug export. Crystals of a selected AcrB–DARPin complex were obtained, and the structure was determined at 2.5-Å resolution. It is the first structure of an integral membrane protein with a selected DARPin molecule binder. The structure reveals a previously unknown asymmetric conformation of the efflux pump, in which each of the three subunits has a unique well-defined conformation. The internal asymmetry of AcrB is underlined by the fact that only two of the three subunits of AcrB are recognized by DARPins in crystallo and in solution and is in contrast to the 3-fold symmetric structures reported to date [6–8]. The structural features described here together with the enhanced resolution allow us to deduce a pathway for drug export and a mechanism for the coupling of substrate export with proton import.

## Results

### DARPin Selection and Inhibitor Screening

DARPin binders were selected in vitro by ribosome display using a designed ankyrin-repeat protein library [11,12]. The selection was performed on purified and in vitro biotinylated AcrB under native conditions. Four rounds of ribosome display were sufficient to select a pool of specific DARPins binding to AcrB. These experiments are to our knowledge the first ribosome display selections performed in the presence of detergent to select specific binders to an integral membrane protein.

The pool of specific binders was subjected to an in vivo screen, based on replica plating, to identify those AcrB-binding DARPins which lead to an inhibitory phenotype. As AcrB is crucial for the transport of certain substrates, its inhibition will consequently lead to accumulation of substrates in the bacterial cell and consequently to cell death. We chose Rhodamine 6G (R6G) as substrate for AcrB. The enriched pool after four rounds of ribosome display was cloned into an expression plasmid under the control of an isopropyl-β-d-thiogalactopyranoside (IPTG)-inducible promoter. E. coli strain XL1-blue was transformed and plated under nonselective conditions, in absence of R6G. About 1,500 colonies were subsequently replica-plated under selective conditions, in the presence of 32 μg/ml R6G. By this procedure, 18 single clones were identified which showed a rhodamine-sensitive phenotype. These selected clones were sequenced and assayed for expression. Analysis of the sequences showed that all 18 clones were different and unique.

The affinities of the AcrB inhibiting DARPins were analyzed by surface plasmon resonance using a BIAcore (http://www.biacore.com) instrument. The analysis was performed on AcrB-coated sensor surfaces with multiple concentrations of the DARPins and was evaluated with a global kinetic fit. The five inhibitors with highest affinity, having dissociation constants in the low nanomolar range (3.6 to 98.9 nM; Figure S1), were chosen for further characterization. Clone 1108_19, which was used for cocrystallization, had a dissociation constant *K_D_* of 28 nM.

### Inhibition Efficiency of the DARPins

**Figure 1:**
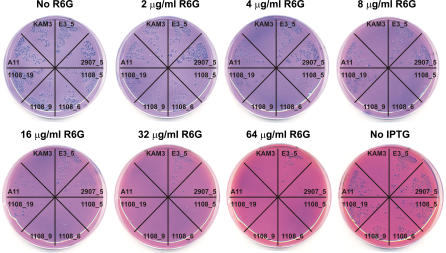
Phenotype of E. coli XL1-Blue Strain Expressing the Selected Inhibitory DARPins Six AcrB inhibitors (2907_5, 1108_5, 1108_6, 1108_9, 1108_19, and A11), one unselected nonbinding DARPin (E3_5), and an *acrB* knockout strain (KAM3 [14], transformed also with E3_5) were ranked for their R6G sensitivity. Induction of the DARPins results in the inhibition of AcrB, yielding an R6G-sensitive phenotype. E. coli cells were transformed with the respective plasmid and plated under different conditions: nonselective, no R6G or no IPTG; selective where different R6G concentrations create an environment in which inhibitory DARPins repress growth. All plates contained IPTG (except plate no IPTG). The plates were colored with Coomassie brilliant blue for better visibility of the colonies.

We rated the inhibition efficiencies of the identified DARPins by spotting the clones onto selective plates with different R6G concentrations, ranging from 2 to 64 μg/ml (Figure 1). All inhibitors showed a significant increase of the sensitivity compared to cells expressing an unselected nonbinding DARPin, termed E3_5 [13]. This control also showed that the expression of a nonbinding DARPin per se has no influence on R6G sensitivity or bacterial growth. The *acrB* gene-deletion strain KAM3 [14] causes hypersensitivity to R6G and was used as a sensitivity control. In absence of R6G, DARPins conferring R6G sensitivity did not influence E. coli growth by themselves. It should be noted, however, that the affinity of the inhibitors does not always correlate with the inhibition efficacy (Figure S1). Possible factors leading to this observation include different expression levels or different binding sites of the DARPins on AcrB. Moreover, the exact inhibition mechanism is not known, even for the inhibitor 1108_19 for which the structure was solved. Different inhibition mechanisms seem plausible ranging from allosteric inhibition of the rotary export mechanism of AcrB by the DARPin to simply preventing the interaction with TolC or AcrA to form the export complex.

### Overall Structure of the AcrB–DARPin Complex: Loss of the 3-Fold Symmetry

**Figure 2:**
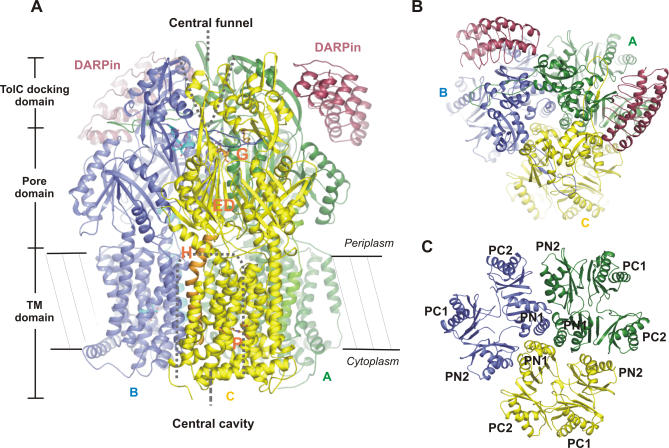
Crystal Structure of the AcrB–DARPin Complex (A) Ribbon diagram of the overall AcrB–DARPin complex structure viewed from the side, depicted in three different colors for each subunit: green, blue, and yellow for A, B, and C, respectively. The DARPins are colored in red. This color code is used throughout all the figures. The locations of the three prominent domains are indicated and the important regions are highlighted and labeled in subunit C (G, gate; ED, external depression; P, putative proton translocation site; H, helix 8). (B) Ribbon diagram of the overall AcrB–DARPin complex viewed from the periplasm. (C) Ribbon diagram of the pore domain viewed as in (B). The subdomains are labeled. All figures of the molecular models were generated with PyMOL [31].

AcrB exists as a homotrimer, with each subunit containing 12 transmembrane (TM) helices and a large periplasmic part formed by two loops between TM helices 1 and 2 and TM helices 7 and 8. The trimer consists of three prominent domains, the TM domain, and parallel to the membrane, the adjacent pore domain and the TolC docking domain (Figure 2A). The TM domain encompasses a large central cavity, 35 Å in diameter, through the membrane which is closed toward the central funnel formed by the TolC docking domain by three helices provided by the PN1 subdomains of the pore domain (Figure 2B and 2C). The export of substrates was proposed directly through this central pore. Several structures of ligand-free AcrB and with bound substrates have been solved at moderate resolution (between 3.5 and 3.8 Å). Yu et al. [7,8] reported structures of AcrB–ligand complexes showing ligand binding to the upper wall in the central cavity and additional substrate binding in the periplasmic domain, compatible with mutational studies, which indicated that discrimination between different toxic substrates occurs in the periplasmic domain, rather than in the TM domain [15,16]. All structures determined to date imply 3-fold symmetry and thus the three AcrB subunits displayed the same conformation.

To analyze the interaction and to characterize the inhibition property of a selected DARPin, we determined the crystal structure of one inhibitor (1108_19) in complex with AcrB. The crystals diffracted to 2.5-Å resolution and belonged to space group P2_1_2_1_2_1_. The structure was solved by molecular replacement using the structure published by Murakami et al. [6] as a search model without using the phases of the DARPin. The results of the data collection and refinement are presented in Table 1.

**Table 1:**
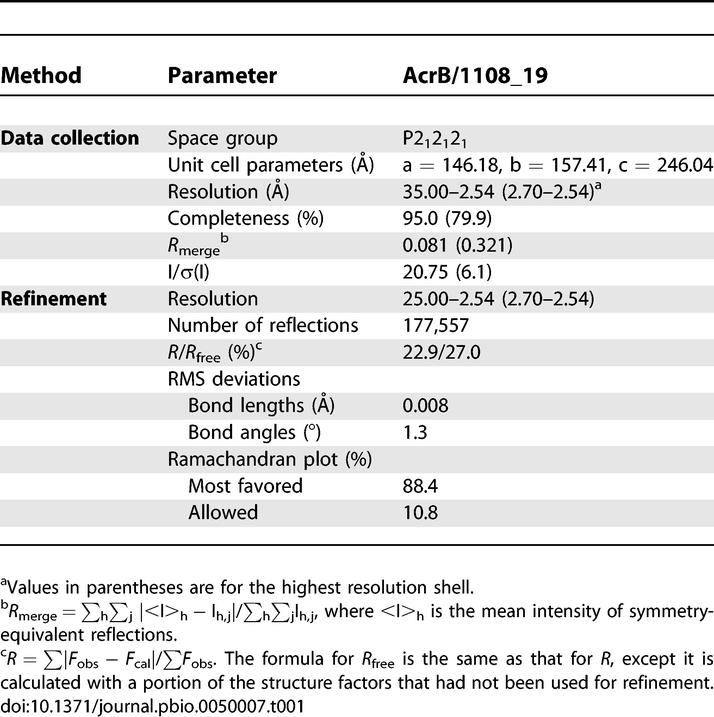
Data Collection and Refinement Statistics

The crystal structure of the AcrB–DARPin complex shows two DARPin molecules bound basically to two subunits, named A and B, in the homotrimeric transporter, while the third subunit, named C, is not bound (Figure 2A and 2B). Unlike previously determined crystal structures, there is no 3-fold symmetry of AcrB with the DARPin molecules stabilizing three distinct conformations of the subunits. The conformations of the DARPin-bound subunits A and B are similar to the known symmetric structure. A superposition on Protein Data Bank entry 1IWG [17] shows a few differences for these two subunits with root-mean-square deviations of 1.72 Å and 1.98 Å, respectively, for 1,033 Cα atoms, compared to 2.95 Å for the third subunit C. To validate in solution the 3:2 (AcrB monomer to DARPin) stoichiometry observed in the crystal structure, we performed sedimentation velocity experiments. Single molecular weight species taking the detergent micelle into consideration were observed, with molecular masses of 374 ± 1.6 kDa for AcrB alone and 408 ± 1.7 kDa for the complex (Figure S2). The mass difference indicates that AcrB in solution is bound by two DARPins, with a calculated mass of 17.8 kDa each.

As can be seen in the crystal structure (Figure 2A and 2B), the DARPins bind mainly to the β-sheet connecting the pore domain with the TolC docking domain interacting, as expected, primarily through their randomized concave surface area. The interface buries a surface of around 1,000 Å^2^ (see Table S1 for detailed interactions). Furthermore, each DARPin interacts with the adjacent preceding subunit of AcrB via one additional hydrophobic interaction involving Leu230 of the intersubunit connecting loop as well as two interactions involving the residues Arg263 and Lys248, again of the preceding subunit (Table S1). Subunit C is in a different orientation, and consequently no DARPin is bound. Notably, the DARPins not only stabilize the asymmetric conformation of AcrB but are also involved in direct interactions in the crystal lattice (Figure S3), resulting in a different crystal form P2_1_2_1_2_1_.

### Structure Reveals Channels Leading through the Individual Subunits

**Figure 3:**
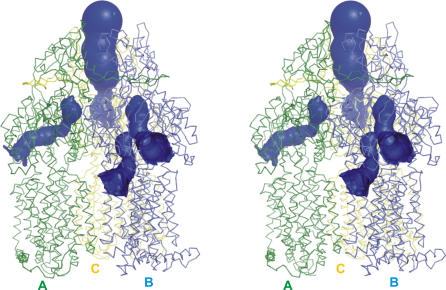
Channels in the AcrB Trimer Stereo view of the channels leading through the periplasmic domain of AcrB. The structure of AcrB is shown as wire model and the DARPins omitted for clarity. The channels are shown as blue surfaces and were calculated using the program CAVER (http://loschmidt.chemi.muni.cz/caver).

**Figure 4:**
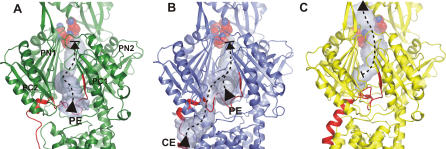
The Extension of the Channels in the Individual Subunits of AcrB The view is the same as in Figure 2A. For simplicity, the DARPins are not shown. The channels are colored in transparent blue. The potential export pathway is represented by dashed lines. The loop forming the bottom of the periplasmic channel entrance (PE) and TM helix 8 are highlighted in red. The gate to the central funnel formed by the residues Gln124, Gln125, and Tyr758 is shown in space filling representation for clarity. (A) In subunit A, the channel is opened to the periplasm, while the gate is in the closed conformation. The pore domain subdomains are labeled. (B) In subunit B, the channel displays an open conformation to the periplasm and to the membrane bilayer (CE). The gate is in a closed conformation. (C) Subunit C displays a closed conformation of the channel entrances, while the gate is open, extending the channel to the central funnel.

The three AcrB subunits are bound in three different conformations, revealing three distinct channels (Figure 3). The width of these channels is sufficient for the passage of typical AcrB substrates. In subunit A, a channel is observed, extending from the external depression through the large periplasmic domain reaching almost the central funnel at the top of the protein (Figure 4A). Here the side chains of residues Gln124, Gln125, and Tyr758 form a gate, closing the channel and therefore preventing direct access to the central funnel. The periplasmic channel entrance corresponds perfectly with the periplasmic binding site indicated in the AcrB–ligand structures of Yu et al. [8]. A similar channel, although a little wider, is present in subunit B (Figure 4B). In addition, the channel is open not only to the periplasm but also to the membrane bilayer at the periphery of the TM domain. In subunit C, the channel entrances are closed due to movements of PC2 and PN1 (Figure 4C). In contrast to subunits A and B, the gate to the central funnel at the top of the periplasmic domain formed by residues Gln124, Gln125, and Tyr758 is now open, allowing the substrate to enter the central funnel from where it most probably reaches TolC and is exported to the cell exterior.

### Structural Rearrangements in the Putative Proton-Translocation Site

**Figure 5:**
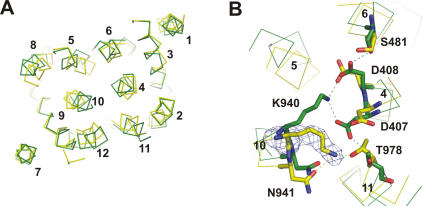
Conformational Changes in the TM Region of AcrB (A) Wire model of the superpositioned TM domains of subunit A and subunit C viewed from the periplasmic side. Subunit B is omitted since it displays a similar conformation as subunit A. The individual helices are labeled. (B) Detailed interactions of the amino acid residues in the putative proton-translocation site viewed in the same orientation as in (A). Residues involved in the hydrogen-bonded network (dashed lines) are labeled. The |F*o*-F*c*| omit electron density map (blue mesh) of Lys940 in subunit C is contoured at 3.5 σ.

The opening and closing of the channel entrances and the gate is coupled to structural rearrangements in the putative proton-translocation site in the TM domain (Figure 5A) involving residues in the middle of the TM helix 4 (Asp407 and Asp408) and TM helix 10 (Lys940), respectively (Figure 5B). These charged residues are localized in the center of the hydrophobic TM domain and have been shown to be essential for the proper function of AcrB, since the mutation of these residues leads to complete loss of drug resistance [18]. In subunits A and B, Lys940 forms a salt bridge to the side chains of Asp407 and Asp408, which are also involved in H-bonds with Ser481 and Thr978 presenting the same conformation described to date [6]. In subunit C, this salt bridge is not formed; instead Lys940 is tilted away, forming new polar contacts to Asn941 and Thr978.

The conformational changes described for the putative proton-translocation site are linked to the opening and closing of the channel entrances and the gate described above via rigid movements of TM helix 5 and TM helix 8 (Figure 5A). Distinct conformational differences of the polypeptide stretch that connects the N-terminal end of TM helix 8 to PC2 can be observed in each subunit (Figure 4). The functional importance of this connection is underlined by the fact that each subunit displays a different conformational state of this polypeptide stretch. In subunit A, the stretch shows a long random coil conformation, whereas in subunit B, it adopts partially a helical conformation but with still some random coil conformation. In subunit C, the polypeptide segment is in a completely α-helical conformation continuing the TM helix 8 up to the headpiece subdomain PC2.

The loss of the interaction of Asp407, Asp408 with Lys940 in the putative site for proton-translocation, the simultaneous closing of the channel entrances, and the opening of the gate are evident. This hints at a coordinated control or a coupling of drug export and proton-translocation. The coupling seems to occur via the polypeptide segment that connects TM helix 8 and PC2.

### Comparison of Asymmetric AcrB Structures

During the review process of this manuscript, the asymmetric structure of AcrB was published by two other groups [19,20]. Our experimental approach, however, differs from theirs as we apply a new technology using DARPins as crystallization aid. This lead to a new crystal form, P2_1_2_1_2_1_, and, finally, crystals diffracting to higher resolution. Notably, the structural comparison reveals that all three independent structure determinations of AcrB resulted all in almost identical structures. The root-mean-square deviations for 1,032 Cα atoms (Table S2 and Video S1) is around 1, which is a very good correlation at the given resolutions of the different structures (2J8S, 2.5 Å; 2DHH, 2.8 Å [19]; 2GIF, 2.9 Å [20]). Importantly, the region where the DARPins bind shows no significant differences in all the structures. This clearly demonstrates that the selected DARPins bind and stabilize an existing conformational state and do not induce a nonnative conformation upon binding. Murakami et al. [19] also crystallized the protein in the presence of two substrates, which greatly substantiates our findings. In conclusion, all three crystal structures lead to virtually identical conclusions about the drug export pathway and we all proposed similar export mechanisms.

## Discussion

Cocrystallization of the membrane protein AcrB and a DARPin inhibitor selected from a large combinatorial library resulted in crystals that diffract to high resolution. The significant higher resolution of our structure compared to the structures published to date allowed unambiguous modeling of the side chain conformations and the interpretation of structural rearrangements extending from the putative proton-translocation sites in the center of the membrane to the gate in the periplasmic domain. The quality of the structure is underlined by 11 resolved detergent molecules (Figure S4). Our work shows for the first time the structure of an integral membrane protein with a selected DARPin molecule, an experimental approach that has great potential for the structural biology of these exceedingly difficult proteins. DARPins may aid by stabilizing certain conformations and by extending the hydrophilic surface similar to antibody-derived protein fragments.

**Figure 6:**
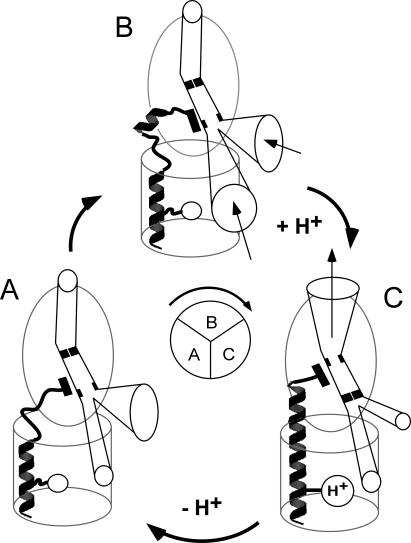
Schematic Drawing of the Transport Pathway of the AcrB Efflux Pump Suggesting a Rotary Mechanism Proposed mechanism: Substrate may bind into the channel through the open entrances of subunit B. Helix 8 is only weakly pronounced and mostly random coil. Changes of the protonation state of residues of the putative translocation site induce the formation of helix 8 and thus the closing of the channel entrances and the simultaneous opening of the gate, extending the channel to the central funnel at the top of the headpiece showing the conformation of subunit C. The bound substrate can be released into the central funnel from where it reaches TolC and finally the cell exterior. Deprotonation takes place, leading to the collapse of helix 8, and the channel goes back to the original conformation (subunit A).

In contrast to the previously known R32 crystal form of AcrB with one monomer in the asymmetric unit, in our structure there are one AcrB trimer and two DARPins in the asymmetric unit, and therefore there are no constraints regarding the conformation of the individual subunits of AcrB. Indeed, the crystal structure shows different conformations for the three individual subunits and reveals channels extending either from the periplasm and the membrane bilayer to a gate near the central funnel at the top of the protein or as in one subunit extending from the gate directly into the central funnel. Moreover, we were able to confirm the 3:2 stoichiometry in solution, confirming the internal asymmetry of AcrB. We believe that the three conformations likely present intermediates in the transport cycle and suggest a rotary mechanism for drug transport where the three subunits strictly alternate their conformation in a concerted way (Figure 6). The R6G-sensitive phenotype of cells expressing the DARPin supports this hypothesis. AcrB may be locked by the DARPins and therefore the rotation of the molecule is stopped. It seems unlikely that the subunits export drugs independent of each other. If this was the case, subunit C would still be functional, and this would be in contrast to the observed inhibitory phenotype induced by the DARPin. Another possibility for the inhibitory effect of the DARPin is that either TolC or AcrA is unable to bind AcrB anymore, resulting in the loss of function of this resistance-nodulation-cell division–type transport protein.

We believe that AcrB operates through a mechanism resembling the alternating-access mechanism which is the substrate-translocation mechanism most commonly described to secondary membrane transporters [21]. We propose that substrates could enter the channel in subunit B via the transmembranal groove at the periphery of the TM domain or via the external depression in the periplasmic part of the structure close to the outer leaflet of the membrane. Substrate would then be specifically bound with the gate of the channel still closed to the central funnel at the top of the periplasmic headpiece. Upon protonation of the proton gating residues Asp407 and/or Asp408, conformational changes in the TM helix 8 take place, which in turn trigger the conformational changes in the periplasmic headpiece. The channel entrances then close through movements of subdomains PN1 and PC2 opening the gate toward the central funnel at the top of the headpiece, releasing the bound substrate into the funnel, from where it could reach TolC and finally the cell exterior.

While this model is based on the interpretation of the structural information seen in the asymmetric structure, it contains all features necessary for the export of substrates and simultaneous import of protons. It is also consistent with mutational studies available to date [8,17]. Further experiments using the crystals of the complex with drug substrates may substantiate this mechanism. In fact, similar to the experiments performed by Murakami et al. [19], we also could show binding of minocycline in the identified channel at 2.5-Å resolution (unpublished data). The structural findings most likely rule out the previously proposed export of substrates directly through the central pore of the structure.

## Materials and Methods

### AcrB production and purification.

AcrB was overexpressed in E. coli strain BL21 (DE3; Novagen, EMD Biosciences, http://www.emdbiosciences.com) overnight at 30 °C using a pET28 vector (Novagen) containing *acrB* with a hexahistidine-tag at the C terminus. Cells of 1-L bacterial culture were disrupted with a French pressure cell, and the membranes were collected and solubilized in buffer A (20 mM Tris-HCl [pH 7.5], 150 mM NaCl, 10 mM imidazole, 10% glycerol), containing 1% (w/v) *n*-dodecyl-β-d-maltoside (DDM) (Anatrace, http://www.anatrace.com). Lipids and debris were removed by ultracentrifugation at 100,000*g* for 1 h. The solubilized protein was purified with affinity chromatography using Ni-NTA (Qiagen, http://www.qiagen.com), equilibrated with buffer A, containing 0.03% DDM. The column was washed using this buffer and 50 mM imidazole, respectively. Purified AcrB was eluted with 200 mM imidazole added to the above buffer.

For biotinylation purposes, AcrB was eluted in 200 mM imidazole (pH 7.5) as the only buffering agent. The protein was chemically biotinylated using a 15 molar excess EZ-Link Sulfo-NHS-LC-Biotin (Pierce Biotechnology, http://www.piercenet.com) for 30 min at 4 °C. Free biotin was removed using a HiTrap Desalting column (5 ml; Amersham Biosciences, http://www.amersham.com) equilibrated in buffer B (20 mM Tris-HCl [pH 7.5], 150 mM NaCl, 0.03% [w/v] DDM).

### In vitro selection by ribosome display.

Four rounds of ribosome-display selection were carried out using an N3C DARPin library [11,12]. In vitro biotinylated AcrB was immobilized on neutravidin-coated wells blocked with BSA. To eliminate BSA- and neutravidin-binding library members as well as further unspecific binders, two pre-panning steps were applied, where neutravidin, BSA, and biotinylated maltose binding protein (MBP) [11] were present. In the actual panning step, binders to AcrB were thus selected. The panning procedure was performed exactly as described previously [11], with the modification that all buffers contained 0.03% (w/v) DDM, to keep AcrB stable and solubilized.

### In vivo selection by replica plating.

The pool of specific binders was subjected to an in vivo screen, based on replica plating, to identify those AcrB-binding DARPins with an inhibitory phenotype. For this purpose, the selected pool of binders was cloned into the vector pQIA [22]. This vector carries an expression cassette for the selected DARPins under the control of an IPTG-inducible T5 promoter.

The pool of binders was transformed into E. coli XL1-blue cells (recA1 endA1 gyrA96 thi-1 hsdR17 supE44 relA1 lac [F′ proAB lacIqZΔM15 Tn10 (Tetr)]; Stratagene, http://www.stratagene.com) and plated under nonselective conditions (ampicillin, IPTG, no R6G). About 1,500 colonies were subsequently replica-plated under selective conditions (ampicillin, IPTG, R6G [32 μg/ml]). By this procedure, 18 N3C clones were identified which showed a rhodamine-sensitive phenotype. The characterization of these DARPins included sequencing, expression tests, surface plasmon resonance, MIC determination, size exclusion chromatography studies, and cocrystallization.

### Drug susceptibility assay.

The susceptibility to R6G of E. coli XL1-blue cells expressing the selected DARPins were determined by sequential 2-fold dilutions with LB agar/glucose plates containing 0.1 mM IPTG. R6G was used at concentrations of 2, 4, 8, 16, 32, and 64 μg/ml. Bacterial growth was examined after 24 h at 37 °C.

### Surface plasmon resonance.

Surface plasmon resonance was measured using a BIAcore 3000 instrument at 20 °C. The running buffer was buffer B. An SA chip (BIAcore) was used with 700 RU of biotinylated AcrB immobilized. The DARPin binding was measured at a flow of 50 μl/min with 5-min buffer flow, 2-min injection of AcrB-binding DARPin in varying concentrations (2.5 nM to 60 nM), and an off-rate measurement of 30 min with buffer flow. The signal of an uncoated reference cell was subtracted from the measurements. The kinetic data of the interaction were evaluated using the program BIAevaluation 3.0 (BIAcore), and global fits were used to determine *K_D_*. The possible avidity effect of the AcrB trimer was not taken into account in the fit.

### Analytical ultracentrifugation.

Sedimentation velocity experiments were conducted using a Beckman ProteomeLab XL-I (http://www.beckmancoulter.com) with an An-50 Ti analytical rotor (Beckman). The samples were equilibrated in buffer B at a concentration of 0.42 mg/ml. All data acquired from this experiment were obtained using the UV/Vis absorbance detection system (Perkin Elmer, http://www.perkinelmer.com) on the ultracentrifuge at 280 nm and double sector 12-mm charcoal-filled Epon centerpieces. The experiment was conducted at 4 °C at a speed of 40,000 rpm. The data of 300 scans were analyzed using the LAMM equation in the program SEDFIT [23].

### Crystal preparation.

To obtain crystals of the AcrB–DARPin complex, AcrB and the selected AcrB-inhibiting DARPin 1108_19 were purified separately as described [12]. A small molar excess of the DARPin with regard to the AcrB monomer was used to prepare the complex, followed by purification on a Tricorn Superdex-200 (Amersham Biosciences) column equilibrated in buffer B. The peak fractions containing AcrB in complex with the DARPin were concentrated using a 30-kDa-cutoff concentrator (Amicon Ultra; Millipore, http://www.millipore.com), exchanged into 10 mM HEPES (pH 7.5), 50 mM NaCl containing 0.03% (w/v) DDM, and again concentrated to 13 to 15 mg/ml.

Initial crystallization screening was done in 96-well, sitting drop crystallization plates (Greiner Bio-One, http://www.greinerbioone.com). The initial crystals were refined using standard techniques. The crystals used for data collection appeared within 4 d in 8% PEG 4000, 50 mM ADA (pH 6.5), 200 mM (NH_4_)_2_SO_4_ in a hanging drop vapor diffusion experiment at 20 °C. For data collection, the crystals were soaked in six steps for about 15 s in the mother liquid containing 5% to 30% glycerol and flash frozen into liquid propane.

### Data collection, phase determination, and structure refinement.

Data were collected at the Swiss Light Source beamline PX (http://sls.web.psi.ch/view.php/about/index.html) and processed using the program XDS [24]. The crystal belonged to space group P2_1_2_1_2_1_, with a Matthews coefficient V_M_ of 3.8 Å^3^/Da, corresponding to an estimated water content of 67.7%.

The crystal structure was solved by molecular replacement using the program PHASER [25,26], with the structure of the AcrB monomer (Protein Data Bank code 1IWG [6]) used as search model. A PHASER search with the DARPin E3_5 (Protein Data Bank code 1MJO [13]) as search model did not yield a meaningful solution. The information obtained from the conventional PHASER protocol for the three AcrB monomers was sufficient to model one DARPin molecule into the resulting electron density with the program O [27]. The second DARPin also was positioned in O. Refinement of the structure was carried out through multiple cycles of manual rebuilding using the program Coot [28] and refinement using CNS [29] resulting in a final model with an *R* factor of 22.9 and an *R*_free_ factor of 27.9. The refined structure of the AcrB–DARPin complex was validated by the program PROCHECK [30]. Three-dimensional structural figures were prepared by using PyMOL [31].

## Supporting Information

Figure S1 Surface Plasmon Resonance Analysis of the AcrB–DARPin Complexes The binding kinetics of selected DARPins were analyzed using a BIAcore instrument. Different concentrations of DARPin (2.5, 5, 8, 15, 25, 40, and 60 nM) were applied to a flow cell with immobilized AcrB for 2 min, followed by washing with buffer flow. The global fit is indicated in the figure by black lines.
(64 KB DOC)

Figure S2 Sedimentation Velocity Analysis of AcrB and the AcrB–DARPin Complex Using the LAMM Equation in the Program SEDFIT (A) AcrB. (B) AcrB–DARPin complex.
(27 KB DOC)

Figure S3 Crystal Packing of AcrB–DARPin Complex Crystals The DARPins (in red) are involved in crystal contacts and thus account for the different space group and most likely for the high resolution of the structure.
(902 KB DOC)

Figure S4 Detergent Molecules in the Structure The structure contains 11 *n*-dodecyl-β-d-maltoside (DDM) detergent molecules underlining the quality of the structure.
(738 KB DOC)

Table S1 Detailed Interactions of the AcrB–DARPin Complex Direct hydrogen bonds between AcrB and the bound DARPins identified by the program LIGPLOT and the interface accessible surface area calculated with the Protein-Protein Interaction Server.
(49 KB DOC)

Table S2 Comparison of the Asymmetric AcrB Structures Root-mean-square deviations of Cα coordinates calculated by the program lsqman.
(41 KB DOC)

Video S1 Comparison of the AcrB–DARPin Complex with Other Asymmetric AcrB Structures Superposition of the Cα atoms of the AcrB–DARPin complex (green) with 2DHH (blue) and 2GIF (pink).
(3.2 MB WMV)

### Accession Numbers

The atomic coordinates and structure factors of the described complex have been deposited in the Protein Data Bank (http://www.rcsb.org) with accession number 2J8S.

## Abbreviations

DARPin: designed ankyrin-repeat protein
IPTG: isopropyl-β-d-thiogalactopyranoside
R6G: rhodamine 6G
TM: transmembrane
