# Supplementary material for: Drug Export Pathway of Multidrug Exporter AcrB Revealed by DARPin Inhibitors
Source: PLoS Biol. 2006 Dec 26;5(1):e7. doi: 10.1371/journal.pbio.0050007 (PMC1717020; doi:10.1371/journal.pbio.0050007)
Supplement: Supplementary file 1 [file pbio.0050007.sg001.doc]

**Figure S1.** Surface Plasmon Resonance Analysis of the AcrB-DARPin complexes

SPR analysis of 1108_19

| **Clone** | **k_on_ [M^-1^s^-1^]** | **k_off_ [s^-1^]** | **K_D_ [M]** | **R6G sensitivity**  **[μg/ml R6G]** |
| --- | --- | --- | --- | --- |
|  |  |  |  |  |
| 1108_19 | 8.7·10^5^ | 2.4·10^-2^ | 28.0·10^-9^ | 32 |
| 1108_05 | 1.2·10^6^ | 6.5·10^-2^ | 52.3·10^-9^ | 8 |
| 1108_06 | 3.4·10^5^ | 3.4·10^-2^ | 98.9·10^-9^ | 16 |
| 1108_09 | 1.1·10^6^ | 4.1·10^-3^ | 3.6·10^-9^ | 4 |
| 2907_05 | 1.8·10^6^ | 4.2·10^-2^ | 23.4·10^-9^ | 32 |
|  |  |  |  |  |
