# Supplementary material for: Drug Export Pathway of Multidrug Exporter AcrB Revealed by DARPin Inhibitors
Source: PLoS Biol. 2006 Dec 26;5(1):e7. doi: 10.1371/journal.pbio.0050007 (PMC1717020; doi:10.1371/journal.pbio.0050007)
Supplement: Supplementary file 2 [file pbio.0050007.sg002.doc]

**Figure S2.** Sedimentation Velocity Analysis of (A) AcrB and (B) the AcrB-DARPin Complex

**(A)**

rmsd = 0.004265

**MW = 374 ± 1.6 kDa**

**(B)**

rmsd= 0.004182

**MW = 408 ± 1.7 kDa**
