# Supplementary material for: Drug Export Pathway of Multidrug Exporter AcrB Revealed by DARPin Inhibitors
Source: PLoS Biol. 2006 Dec 26;5(1):e7. doi: 10.1371/journal.pbio.0050007 (PMC1717020; doi:10.1371/journal.pbio.0050007)
Supplement: Supplementary file 3 [file pbio.0050007.sg003.doc]

**Figure S3.** Crystal Packing of AcrB-DARPin Complex Crystals
