# Supplementary material for: Drug Export Pathway of Multidrug Exporter AcrB Revealed by DARPin Inhibitors
Source: PLoS Biol. 2006 Dec 26;5(1):e7. doi: 10.1371/journal.pbio.0050007 (PMC1717020; doi:10.1371/journal.pbio.0050007)
Supplement: Supplementary file 4 [file pbio.0050007.sg004.doc]

**Figure S4.** Detergent Molecules in the Structure
