# Supplementary material for: Drug Export Pathway of Multidrug Exporter AcrB Revealed by DARPin Inhibitors
Source: PLoS Biol. 2006 Dec 26;5(1):e7. doi: 10.1371/journal.pbio.0050007 (PMC1717020; doi:10.1371/journal.pbio.0050007)
Supplement: Supplementary file 5 [file pbio.0050007.st001.doc]

**Table S1**

| Subunit A | DARPin E | Subunit C | Distance  (Å) | Subunit B | DARPin D | Subunit A | Distance  (Å) |
| --- | --- | --- | --- | --- | --- | --- | --- |
| Hydrogen bonds (< 3.3 Å) | | | | Hydrogen bonds (< 3.3 Å) | | | |
| Asp^723^ (O) | Arg^23^ (NH2) |  | 3.15 | Tyr^811^ (OH) | Asp^44^ (OD2) |  | 3.12 |
| Tyr^811^ (N) | Tyr^56^ (OH) |  | 3.03 | Tyr^811^ (N) | Tyr^56^ (OH) |  | 3.07 |
| Trp^809^ (NE1) | Thr^78^ (OG1) |  | 2.74 | Ser^802^ (O) | Lys^144^ (NZ) |  | 2.71 |
| Ser^807^ (N) | Asn^112^ (OD1) |  | 2.81 | Ser^805^ (O) | Lys^144^ (NZ) |  | 3.26 |
| Ser^805^ (O) | Lys^144^ (NZ) |  | 2.98 |  |  |  |  |
| Ser^802^ (O) | Lys^144^ (NZ) |  | 2.66 |  |  |  |  |
| Glu^734^ (OE2) | Lys^147^ (NZ) |  | 2.65 |  |  |  |  |
|  |  |  |  |  |  |  |  |
|  | Ile^154^ (O) | Arg^263^ (NH2) | 3.28 |  | Asn^155^ (O) | Arg^263^ (NH2) | 2.67 |
|  | Asn^155^ (O) | Arg^263^ (NH1) | 2.68 |  |  |  |  |
|  |  |  |  |  |  |  |  |
| Interface ASA (Å^2^) | | | | Interface ASA (Å^2^) | | | |
| 921.06 | 1003.48 |  |  | 1057.49 | 1154.29 |  |  |
| Planarity |  |  |  | Planarity |  |  |  |
| 1.78 | 2.02 |  |  | 1.54 | 2.02 |  |  |
