# Supplementary material for: Drug Export Pathway of Multidrug Exporter AcrB Revealed by DARPin Inhibitors
Source: PLoS Biol. 2006 Dec 26;5(1):e7. doi: 10.1371/journal.pbio.0050007 (PMC1717020; doi:10.1371/journal.pbio.0050007)
Supplement: Supplementary file 6 [file pbio.0050007.st002.doc]

**Table S2**

| *2J8S* | *2DHH* [29] | *2GIF* [30] | *1IWG* [6] |
| --- | --- | --- | --- |
| **All (residues 2-1033)** | | | |
| **All** | 1.001 Å (3054 atoms) | 0.806 (3096) | 2.423 (3009) |
| **Chain A** | 0.998 (1018) | 0.835 (1032) | 1.728 (1003) |
| Chain B | 0.982 | 0.735 | 1.981 |
| Chain C | 0.914 | 0.729 | 2.951 |
| **TM (residues 2-28, 330-557, 874-1033)** | | | |
| **All** | 1.063 (1203) | 0.785 (1245) |  |
| **Chain A** | 1.074 (401) | 0.719 (415) |  |
| Chain B | 0.955 | 0.670 |  |
| Chain C | 0.993 | 0.831 |  |
| **Pore domain (residues 38-134, 135-181, 274-330, 567-673, 674-724, 813-859)** | | | |
| **All** | 0.913 (1218) | 0.700 (1218) |  |
| **Chain A** | 0.846 (406) | 0.694 (406) |  |
| Chain B | 0.956 | 0.689 |  |
| Chain C | 0.903 | 0.644 |  |
| **TolC docking domain (residues 182-273, 725-812)** | | | |
| **All** | 0.824 (540) | 0.512 (540) |  |
| **Chain A** | 0.711 (180) | 0.495 (180) |  |
| Chain B | 0.647 | 0.425 |  |
| Chain C | 0.678 | 0.473 |  |
